# Influence of Processing Techniques on Microstructure and Mechanical Properties of a Biodegradable Mg-3Zn-2Ca Alloy

**DOI:** 10.3390/ma9110880

**Published:** 2016-10-28

**Authors:** Pavel Doležal, Josef Zapletal, Stanislava Fintová, Zuzanka Trojanová, Miroslav Greger, Pavla Roupcová, Tomáš Podrábský

**Affiliations:** 1 Faculty of Mechanical Engineering, Institute of Materials Science and Engineering, Brno University of Technology, Technická 2, Brno 616 69, Czech Republic; zapletal@fme.vutbr.cz (J.Z.); roupcova@fme.vutbr.cz (P.R.); podrabsky@fme.vutbr.cz (T.P.); 2 Institute of Physics of Materials, Academy of Sciences of the Czech Republic v. v. i., Žižkova 22, Brno 616 62, Czech Republic; fintova@ipm.cz; 3 Department of Physics of Materials, Faculty of Mathematics and Physics, Charles University in Prague, Ke Karlovu 5, Praha 121 16, Czech Republic; ztrojan@met.mff.cuni.cz; 4 Department of Materials Forming, Faculty of Metallurgy and Materials Engineering, VŠB—Technical University of Ostrava, 17. listopadu 15, Ostrava 725 29, Czech Republic; miroslav.greger@vsb.cz

**Keywords:** biodegradable magnesium alloy, Mg-Zn-Ca, squeeze casting, ECAP processing, microstructure, mechanical properties

## Abstract

New Mg-3Zn-2Ca magnesium alloy was prepared using different processing techniques: gravity casting as well as squeeze casting in liquid and semisolid states. Materials were further thermally treated; thermal treatment of the gravity cast alloy was additionally combined with the equal channel angular pressing (ECAP). Alloy processed by the squeeze casting in liquid as well as in semisolid state exhibit improved plasticity; the ECAP processing positively influenced both the tensile and compressive characteristics of the alloy. Applied heat treatment influenced the distribution and chemical composition of present intermetallic phases. Influence of particular processing techniques, heat treatment, and intermetallic phase distribution is thoroughly discussed in relation to mechanical behavior of presented alloys.

## 1. Introduction

Magnesium and its alloys belong to progressive materials increasingly applied in the transportation, electronic and optical industries. Over the last decades, a new application was found also in the biomedical field. Important advantages predetermining magnesium alloys to be chosen for many usages are their low density, high specific strength and stiffness, and high damping capacity [1,2,3,4,5]. Magnesium alloys have also excellent biomechanical properties for many applications and biocompatibility which allow adopting them as materials convenient for implants in the orthopedics and trauma therapy [2,3,4,6,7,8]. The most restrictive disadvantages of magnesium and its alloys are poor formability and limited ductility at room temperature, which are a consequence of their hexagonal close-packed (hcp) structure with the restricted number of available easy-slip systems and presence of intermetallic phases [9]. Another disadvantage is the quite poor corrosion resistance; however, this disadvantage may be turned into an advantage for biomedical applications like temporary implants [9].

Properties of magnesium alloys are dependent on their chemical composition, [5,6,7,10], mainly on the content and combination of alloying elements. Some of these elements (RE metals, Al, etc.) improving mechanical properties and corrosion resistance of magnesium alloys are toxic, so their content should to be strictly controlled [1,5,10]. Producing technologies and/or additional thermo-mechanical treatment represent another way how to improve the mechanical and corrosion properties of the material [1,5,11]. Production technologies influence the microstructure heterogeneity such as casting defects, distribution of intermetallic phases, grain size and further texture or dislocation substructure. Additional thermo-mechanical treatment may homogenize and refine the microstructure (grain size, size of intermetallic compounds typically present in magnesium alloys, dissolution of intermetallic particles). In spite of the several previous studies concerning the mechanical and corrosion properties of Mg-Zn-Ca alloys, there are still problems regarding optimal chemical composition and thermo-mechanical treatment to achieve the best combination of mechanical and corrosion properties. Chemical composition of the newly developed Mg-3Zn-2Ca alloy presented in the study has been chosen analyzing the literature data. Zinc has great potential as the alloying element for the improvement of mechanical properties such as tensile strength, elongation to fracture and corrosion resistance of Mg alloys [4,6,7,9,11,12,13,14]. The zinc content positively influences the harmfully corrosive effect of Fe and Ni, which are usually present in a small amount in Mg alloys [1,15]. Addition of other alloying elements such as Mn and Ca may even improve mechanical properties of the Mg-Zn system [4,7,15,16,17]. Zn element is also often the main component of various enzymes present in the human body [6]. This fact predetermines Mg-Zn alloys for biomedical applications. However, higher content of Zn decreases the corrosion resistance of Mg-Zn-Ca alloys due to an increase of the corrosion current owing to the corrosion potential rise [4].

The appropriate content of Ca in Mg alloys may improve both the corrosion resistance and mechanical properties; Ca is also incorporated in the human bones and it can accelerate their growth [6]. Ca addition into a magnesium alloy improves the oxidation resistance due to formation of protective oxide layers [18]. It also increases hardness and the creep resistance due to formation of the Mg_2_Ca intermetallic compound with a high melting point [18]. Mg-Ca alloys containing 0.6–1.0 wt % of Ca provide acceptable mechanical and corrosion properties; therefore, the Mg-Ca binary alloys are considered biodegradable materials [6,19]. Magnesium alloys with 1.0–3.0 wt % of Ca are still non-toxic; however, the higher Ca content deteriorates mechanical characteristics and the alloy corrosion resistance [19,20].

Presence of fine particles in the matrix of Mg-Ca-Zn alloys (mainly Mg_6_Ca_2_Zn_3_ and Mg_2_Ca) results in high strength, good creep resistance and acceptable hardness. Mg-Zn-Ca alloys are interesting materials for many applications [9,15,18,21,22,23,24]; nevertheless, the quite low fracture toughness should to be taken into account before use. Formation of the Mg_6_Ca_2_Zn_3_ secondary phase increases tensile strength in the Mg-Zn-Ca alloys [15]. The combination of Zn and Ca solute atoms influences not only mechanical characteristics but also corrosion resistivity [17]. An addition of 2 wt % Zn to the Mg-3Ca alloy improves the mechanical properties (ultimate tensile strength by 23% and ductility by 120%), and also the corrosion resistance may be improved due to the present eutectic phase (Mg + Mg_2_Ca + Ca_2_Mg_6_Zn_3_) [6].

Both the amount and dispersion of present intermetallic phases influence the alloy mechanical properties. Zhang et al. presented in [4] mechanical characteristics of Mg-xZn-1Ca alloy depending on Zn content in a wide range of concentrations from 0 to 6 wt % and 1 wt % of Ca. By addition of the different Zn content levels to the Mg-xZn-1Ca alloy, the ultimate tensile strength varies from 105 ± 4 to 182 ± 5 MPa (Mg-0Zn-1Ca vs. Mg-4Zn-1Ca), the yield stress may vary from 39 to 67 MPa (Mg-0Zn-1Ca vs. Mg-6Zn-1Ca) and the values of elongation to fracture exhibited values from 4.1% ± 0.5% to 9.1% ± 2.5% (Mg-0Zn-1Ca vs. Mg-4Zn-1Ca). 

Wan et al. studied in [19] the influence of Ca in Mg-Ca alloys on mechanical and corrosion properties. The bending and compressive strength was increased with the increase of Ca content from 0.6 to 2.0 wt %. However, they also observed the negative influence of the increasing volume fraction of the Mg_2_Ca phase on the corrosion resistance. The Mg-4.0Zn-0.2Ca alloy examined by Sun et al. in [17] exhibits quite low strength which could be improved by the following hot extrusion. The extruded alloy with fine grains (the average grain size of 3–7 μm) exhibits the tensile yield stress of 240 ± 5 MPa, ultimate tensile strength of 297 ± 5 MPa and elongation to fracture of 21.3% ± 3.0%. 

Many studies (see, for example, [6,9,13,16,19,20,23,25]) are focused on the influence of individual alloying elements on microstructural, mechanical and corrosion characteristics of Mg-Zn-Ca alloys, but only very few of the available sources give any information concerning the synergetic impact of alloying elements and processing technology on the resulting microstructure and mechanical properties.

One of the production methods of magnesium alloys is the gravity casting, even though this process is often accompanied by the presence of casting defects [26]. Squeeze casting technique (SC) is the metal forming process where the solidification is promoted under the high-pressure application within a reusable tool. Molten metal during the SC process is solidified under the applied pressure, which results in the fine-grained microstructure with the excellent surface finish and practically no porosity. Mechanical properties of the SC components can be positively influenced compared to the gravity cast and die cast ones [26]. The SC technique was described in the literature in several modifications (from completely solidified alloys to SC of semisolid metal) [27,28]. With the semisolid melt in the SC process, a positive influence on the tensile properties and elongation to fracture, especially when the SC is followed by some thermal treatment, can be observed.

The present paper deals with Mg-3Zn-2Ca alloy developed for biomedical applications processed by various means. It offers the complex study of the microstructure evolution and resulting mechanical properties. Chemical composition of the Mg-3Zn-2Ca alloy was chosen to reach optimum ratio between strength and plasticity. The Mg-3Zn-2Ca alloy was squeeze cast in the liquid and semisolid states. Thermal treatment was applied with the aim of improving the deformation properties of the alloy. The ECAP treatment applied in the combination with a thermal treatment resulted in the significant improvement of the strength of the gravity cast alloy. 

## 2. Experimental Material and Methods

Gravity cast Mg-3Zn-2Ca (2.5–3.5 wt % Zn and 1.5–2.5 wt % Ca. balance Mg) alloy was used in this study as the default material. Four material states were studied: gravity cast alloy (GC), squeeze cast liquid alloy (SCL), squeeze cast semisolid alloy (SCS) and equal channel angular pressed alloy (ECAP). 

Microstructural analysis of the experimental material was performed in the scanning electron microscope (SEM) Philips XL-30 (Brno, Czech Republic) equipped with the EDS EDAX detector. Specimens for the microstructural evaluation were prepared by the standard metallographic procedures including grinding (SiC papers no 1200, 2500 and 4000) and polishing (diamond paste with grains of 3, 1 and 0.25 μm) using isopropanol as a cooling medium. To reveal the microstructure of the prepared materials, an etchant consisting of 2% Nital was used. The average grain size was estimated by the line method using software for image analysis. 

Tensile tests were performed to obtain basic mechanical properties of Mg-3Zn-2Ca alloy processed by different methods. Mechanical tests were carried out in a Zwick Z250 PC-controlled testing device (Zwick GmbH & Co.KG in Germany, Ulm, Germany) with an initial strain rate of $\dot{\varepsilon }$ = 2.5 × 10^−4^ s^−1^ at room temperature (23 ± 2 °C). Tensile specimens were machined and tested according to the EN ISO 6892-1 standard [29]. The gauge length and diameter of the cylindrical specimens were 25 mm and 5 mm, respectively. Tested samples were cut from the treated semi products so that the longitudinal sample axis was identical with the applied force direction in the manufacturing tool. The specimens for compression tests exhibited diameter and length of 8 mm and 12 mm. For the compression tests, the grip heads were lubricated by MoS_2_. The obtained data are presented in the true stress–true plastic strain curves plot. The yield stress σ_02_ was estimated as the flow stress at the plastic strain of ε = 0.002; ultimate tensile/compression strength σ_UTS_/σ_UCS_ was taken as the true maximum stress achieved in the mechanical test.

The microstructural features and the secondary phase composition were characterized by the X-ray powder diffraction (XRD) by the SmartLab (Rigaku, Tokyo, Japan) with the conventional Bragg-Brenato geometry. The CuKα_1,2_ radiation with the β-filter in a secondary beam and the linear positional sensitive detector D-Tex was used for the microstructure investigations in an angle range of 10°–125°, whit step 0.02° and speed 0.3 s/step. The HighScore Plus program (PANAnalytical, Almelo, The Netherlands) equipped by the JCPDS PDF-4 database (NIST) was used for the qualitative and quantitative analysis of the obtained data. 

The squeeze casting method in the liquid state (SCL) and modified squeeze casting method in the semisolid state (SCS) were applied to refine the original as-cast alloy microstructure, formed in the gravity cast ingots. The cylindrical specimens with a diameter of 40 mm, covered by a protecting stainless steel shell, were heated to a temperature of 720 °C for liquid and to 650 °C for the semisolid casting for 30 min. Specimens were subsequently squeezed at a pressure of 150 MPa for 5 min. The shell of the produced specimens was removed after the squeeze casting process. Specimens for mechanical testing were prepared from an SCL and SCS semi-products with a final diameter of 30 mm.

The equal channel angular pressing was performed in an NMT 3000 type machine (RAKOVNICKÉ TVÁŘECÍ STROJE s. r. o., Rakovník, Czech Republic) with a die angle of 90°. The tool was preheated to a temperature of 350 °C then the sample was passed through the die with a speed of 4 mm·s^−1^. Only one pass through the die channel was applied. Deformation force used for one applied pass through the ECAP die was 3 MN and the cross-section of the final product was of 20 × 20 × 120 mm^3^. To obtain compact products by the ECAP, it was necessary to use backpressure. The pressure of the oil in the system used as the backpressure to the ECAP was 0.5 MPa. 

The heat treatment (HT) was applied in all four states with the aim of obtaining finer microstructure and improved mechanical properties. The HT consisted of heating to a temperature of 450 °C for 24 h followed by quenching in water at an ambient temperature (23 ± 2 °C).

## 3. Microstructure Characteristics

### 3.1. Gravity Cast Alloy (GC)

**Figure 1:**
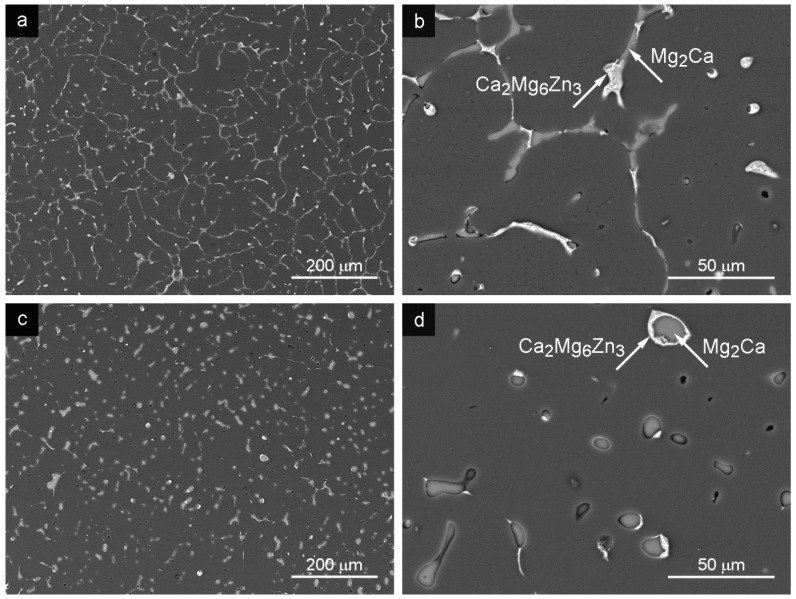
Microstructure of the GC alloy: (**a**) as-cast; (**b**) secondary phases in the as-cast state; (**c**) GC + HT; (**d**) secondary phases in GC + HT state depicted in the SEM–BSE mode.

The microstructure of the GC alloy consisted of α grains (solid solution of alloying elements in Mg), decorated by second phase particles at the grain boundaries (Figure 1a,b). The average grain size of the GC alloy was measured to be 40.7 ± 5.9 μm (Table 1). Two different intermetallic phases were identified in the microstructure presented in Figure 1b. Based on the phase analysis results, introduced in Table 2, Mg_2_Ca intermetallic phase was identified, in the alloy microstructure (see Figure 1b), typical for Mg-Ca systems [8]. The second intermetallic phase present in the microstructure, identified based on the chemical composition measured by the EDX system (see Table 2 and literature data [8]), was Ca_2_Mg_6_Zn_3_ intermetallic compound (light particles in Figure 1b). 

Optimized HT affected only the intermetallic particles. The average grain size of CG alloy was not affected by the applied HT. The HT partially dissolved particles of both types. The Mg_2_Ca phase was reshaped into spheres surrounded by a thin shell of the Ca_2_Mg_6_Zn_3_ intermetallic phase, as it is demonstrated in Figure 1c,d. Applied HT changed also the chemical composition of the matrix (hcp Mg) and present intermetallic phases as it is documented in Table 2. Significant influence of the HT was observed in the case of the Ca_2_Mg_6_Zn_3_ phase. Observed decrease of Zn and Ca content in the Ca-Mg-Zn phase, due to its partial dissolution during the HT was observed. Zn atoms from the Ca_2_Mg_6_Zn_3_ phase diffused into the α solid solution while the Ca atoms were absorbed into the Mg-Ca phase (Table 2). Observed dissolution of intermetallic phases was manifested by the increase of solute elements in the alloy matrix (Table 2).

### 3.2. Squeeze Cast Liquid Alloy (SCL)

**Figure 2:**
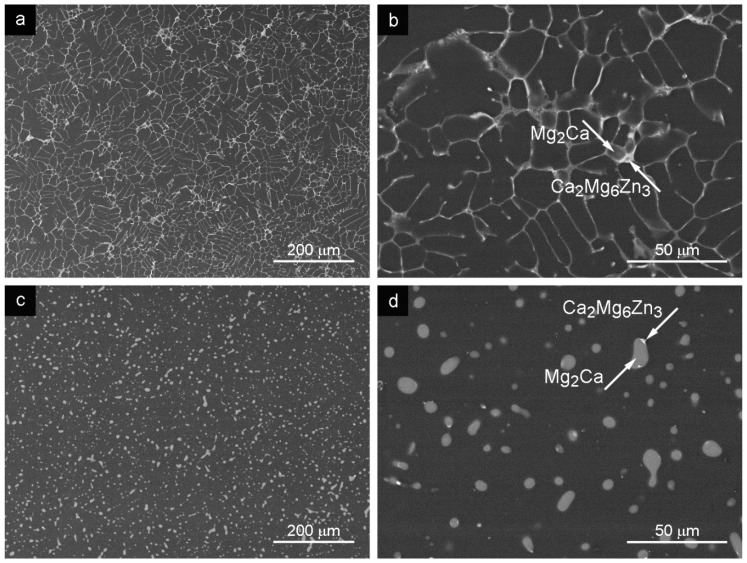
Microstructure of SCL sample: (**a**) SCL state; (**b**) secondary phases in the SCL state; (**c**) SCL + HT; (**d**) secondary phases in SCL + HT depicted in the SEM–BSE mode.

The microstructure of the SCL alloy consisted of fine grains of the α solid solution and intermetallic phases creating a network between the grains (Figure 2a,b). The average grain size of the SCL alloy, 9.6 ± 0.7 μm, was much lower compared with the GC material as it can be seen from Table 1. Two types of intermetallic phases were present in the microstructure, as it follows from Figure 2b, namely Mg_2_Ca and Ca_2_Mg_6_Zn_3_, determined by their chemical composition introduced in Table 2, and the phase composition was determined by the X-ray analysis (described in Chapter 3.5). Only a small amount of the Ca_2_Mg_6_Zn_3_ phase was observed in the microstructure mainly situated at the interface between the matrix grains and Mg_2_Ca phase (see Figure 2b).

The continuous network of the second phase particles partially dissolved after HT and changed shape into globular particles (Figure 2c,d). A small amount of the remaining Ca_2_Mg_6_Zn_3_ phase was present at the interface between spheroid Mg_2_Ca phase and the matrix as it is indicated in Figure 2d. Similar partial dissolution and absorption of Ca_2_Mg_6_Zn_3_ phase as well as in the GC sample after the HT was observed. Zn from the Ca_2_Mg_6_Zn_3_ phase was dissolved and absorbed by the matrix (see Mg content in Table 2) and, similarly, Ca atoms were absorbed by the Mg_2_Ca phase, which was also proven by the chemical analysis (Table 2). 

### 3.3. Squeeze Cast Semisolid Alloy (SCS)

**Figure 3:**
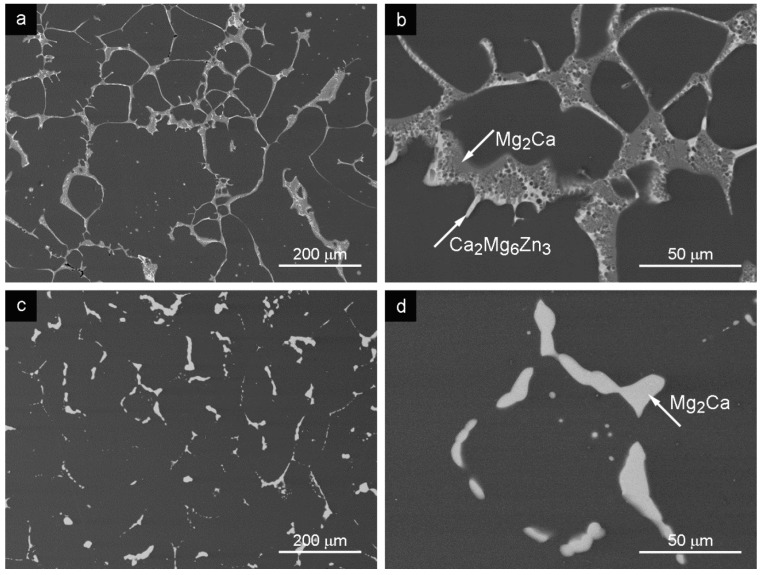
Microstructure of the SCS sample: (**a**) SCS state; (**b**) secondary phases in SCS; (**c**) SCS + HT; (**d**) secondary phases in SCS + HT state depicted in the SEM–BSE mode.

The resulting microstructure, obtained after modified squeeze casting in the SCS state, is shown in Figure 3a. The network of the secondary phase was observed at the grain boundaries. Two types of intermetallic phases were present in the microstructure, namely Mg_2_Ca and Ca_2_Mg_6_Zn_3_ (Table 2 and Figure 3b), similar to the GC and SCL case. The grain size distribution is bimodal. The large grains (~200 μm) of the solid solution containing small intermetallic particles were observed in the microstructure as well as fine grains with the grain size of 9.6 ± 0.7 μm (see Figure 3a, Table 1). 

**Figure 4:**
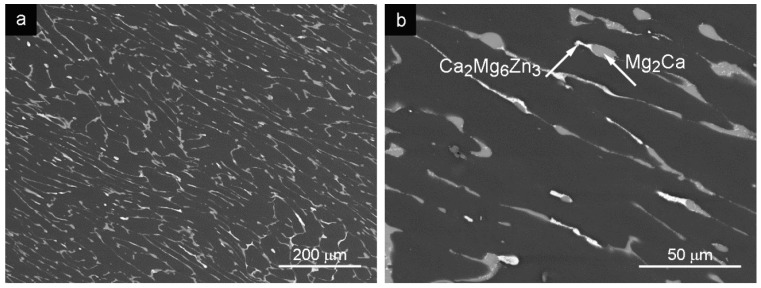

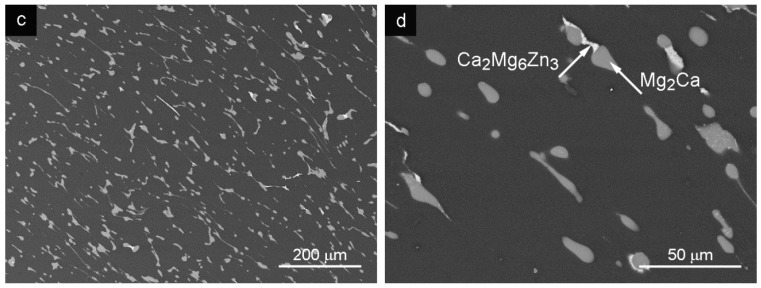
Microstructure of the ECAPed alloy: (**a**) ECAPed state; (**b**) secondary phases in the ECAPed state; (**c**) HT + ECAP; (**d**) secondary phases in HT + ECAP state depicted in the SEM–BSE mode.

HT of the alloy in SCS state resulted in the dissolution of the present intermetallic phases (Figure 3c,d). In the microstructure of SCS alloy after the HT, no Ca_2_Mg_6_Zn_3_ phase particles were observed. The Ca_2_Mg_6_Zn_3_ phase was completely dissolved during the HT and absorbed by other structural components. Only rounded Mg_2_Ca particles, situated in the grain boundaries, were observed in the microstructure (Figure 3d). The remaining Mg_2_Ca phase particles became larger compared with the particles estimated after the heat-treated GC and SCL states as is demonstrated in Figure 1c,d and Figure 4c,d. Also in the case of the SCS alloy after HT, the chemical composition, shown in Table 2, indicates the absorption of Zn and Ca atoms from the dissolved Ca_2_Mg_6_Zn_3_ phase into the matrix and the Mg_2_Ca phase respectively.

### 3.4. ECAPed Mg-3Zn-2Ca (Equal Channel Angular Pressing)

ECAP processing of the alloy in the GC state resulted in the microstructure containing elongated grains oriented into extrusion direction surrounded by the intermetallic phases present at the grain boundaries (Figure 4a,b). The average grain size (Table 1) in the extrusion direction was 84.3 ± 0.3 μm. The average grain size in the direction perpendicular to the processing axis was 30.5 ± 2.6 μm. Chemical composition of the microstructural features was not affected by higher temperature in the preheated ECAP tool. The present Ca_2_Mg_6_Zn_3_ phase was observed at the interface between Mg_2_Ca phase and the matrix (Figure 4b).

HT of the GC alloy resulted in the fine microstructure shown in Figure 1c,d. The following ECAP treatment deformed the grain structure toward the extrusion direction (Figure 4c,d). The present Ca_2_Mg_6_Zn_3_ phase was observed in the interface between the Mg_2_Ca phase and the matrix (Figure 4d).

### 3.5. Chemical and Phase Composition of Mg-3Zn-2Ca Alloy Processed by Various Techniques

**Figure 5:**
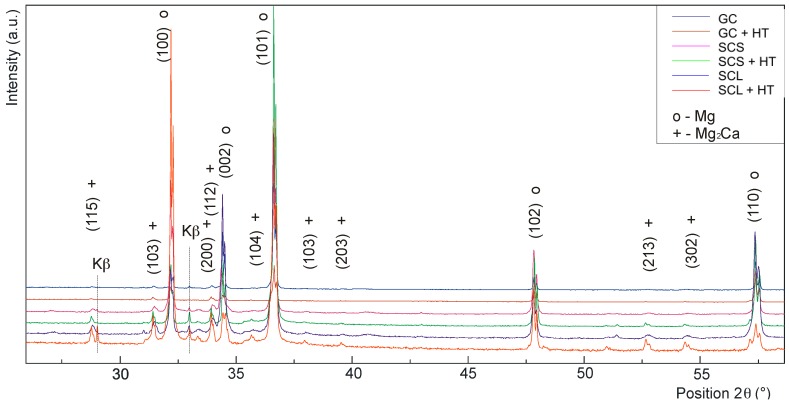
X-ray pattern of Mg-3Zn-2Ca magnesium alloy processed by different methods.

Chemical composition of the observed intermetallic phases in the alloy processed by different technologies was modified due to the heat treatment in all the cases (Table 2). The X-ray pattern of Mg-3Zn-2Ca alloy processed by different methods is shown in Figure 5. The phase composition of the matrix (α grains) and the present intermetallic phases was similar for all the alloy states after applied HT. Only in the case of the SCS + HT alloy state was the Ca_2_Mg_6_Zn_3_ phase completely dissolved and absorbed by other structural components during the heat treatment (matrix and the Mg_2_Ca phase) (Table 2, Figure 3c,d). The phase composition of the alloy was not changed by ECAP treatment compared to the GC state. 

## 4. Deformation Characteristics

**Figure 6:**
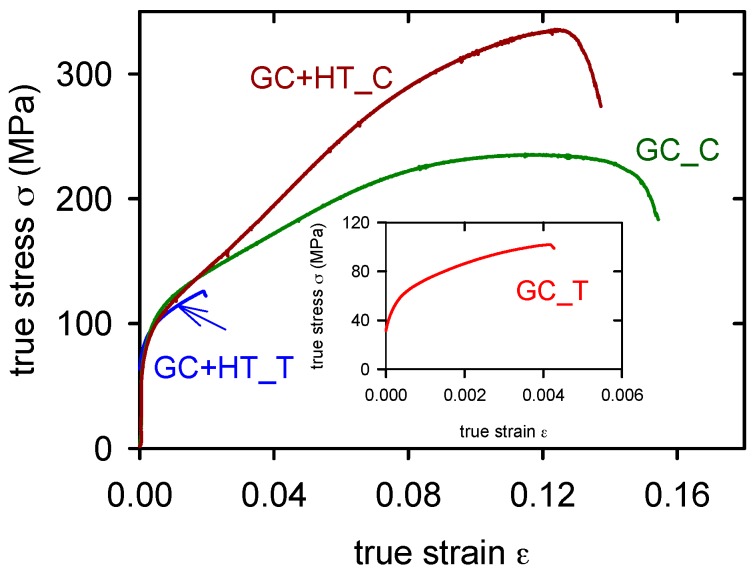
True stress–true strain curves obtained for GC alloy in tension (T) and compression (C). Due to very low ductility of the GC alloy in tension, the curve is introduced in the insert.

True stress–strain curves obtained at room temperature in tension and compression for the GC alloy are introduced in Figure 6. Characteristic values—the yield stress, σ_02_, the ultimate tensile/compression strength σ_UTS_/σ_UCS_, strain to fracture, ε, were evaluated and are presented in Table 3 and Table 4. All mechanical characteristics were estimated with the standard deviation of ±5%.

**Figure 7:**
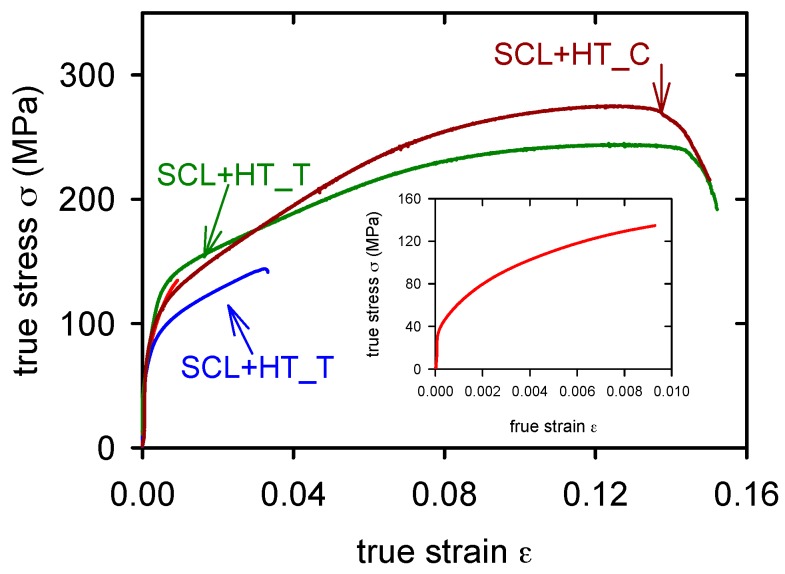
True stress–true strain curves obtained for SCL alloy in tension (T) and compression (C). Due to very low ductility of the as cast alloy in tension, the curve is introduced in the insert.

**Figure 8:**
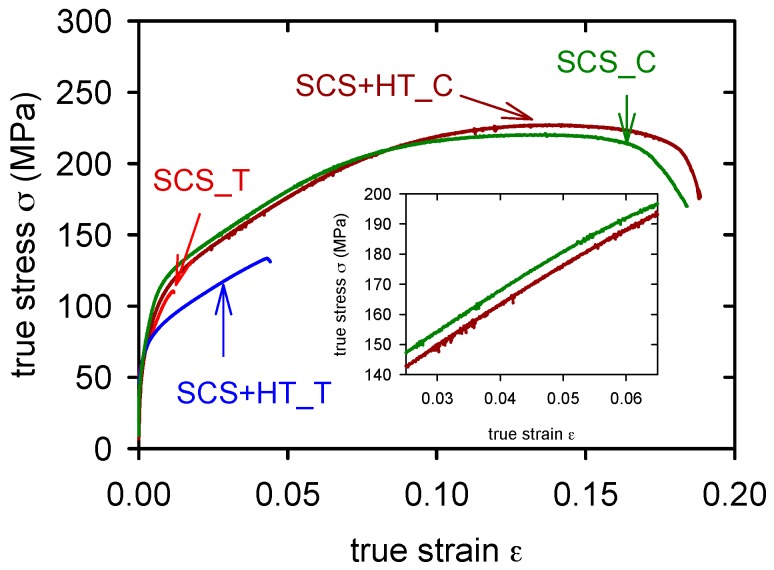
True stress–true strain curves obtained for SCS alloy in tension (T) and compression (C). Serrated flow estimated in compression is obvious from the insert.

From Figure 6 it is obvious that the ductility of the GC alloy is very low in tension, lower than 1% (see insert in the Figure 6). Applied heat treatment increased the plasticity in tension up to 2%, while in the compression test it remained approximately the same. The yield stress obtained in compression is slightly lower than that found in tension. Similar curves were found for SCL alloy and SCL + HT alloy, Figure 7. SCL alloy showed lower yield stress and slightly higher ductility than measured in the GC alloy. The pressure application during the squeeze casting process of the semisolid alloy (SCS) decreased the tensile yield stress (see Table 3). Corresponding values obtained in compression are slightly higher (see Table 4). True stress–strain curves obtained in tension and compression are introduced in Figure 8 for SCS and SCS + HT alloys. Small serrations were observed on curves received in compression. These serrations are more pronounced in the alloy after heat treatment. 

**Figure 9:**
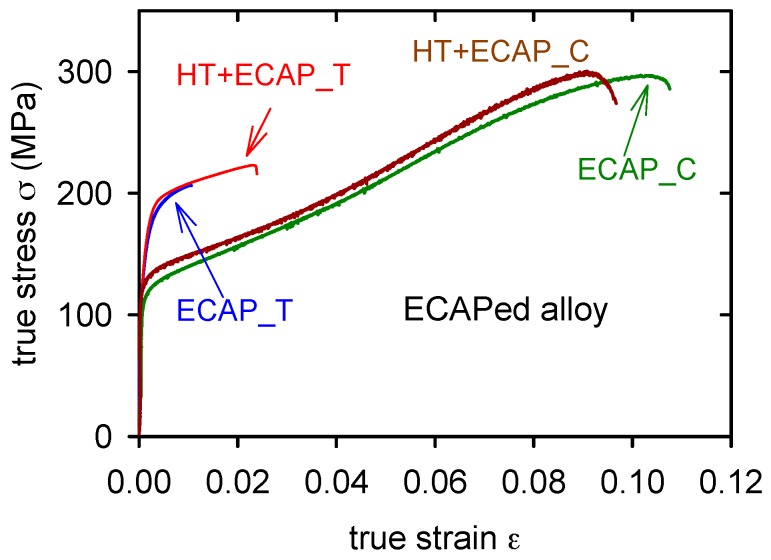
True stress–true strain curves obtained for ECAPed alloy and HT + ECAPed alloy in tension (T) and compression (C).

**Figure 10:**
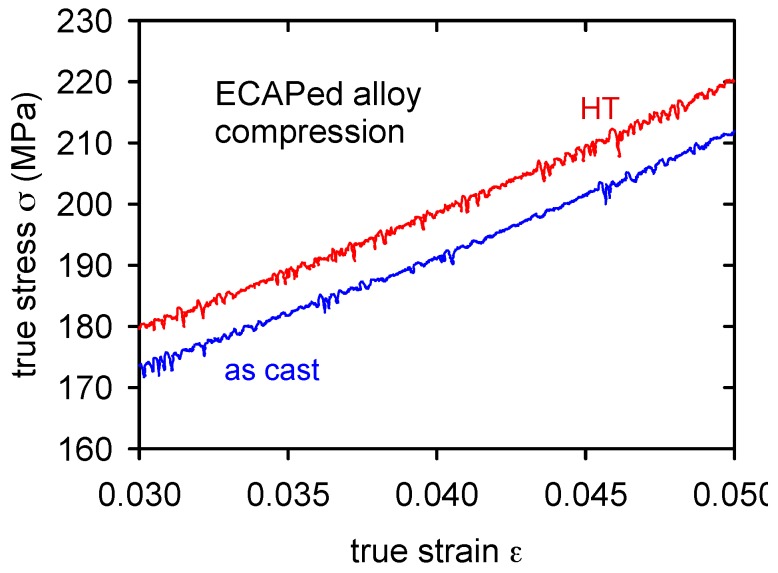
Serrated flow estimated for the ECAPed alloy during deformation in compression.

The stress–strain curves of ECAPed alloy and HT + ECAPed alloy are introduced in Figure 9. Significant difference was found between the yield stresses in tension and compression (see Table 3 and Table 4). Serrated flow was found in both compressive curves ECAP and HT + ECAP as is demonstrated in Figure 10. 

## 5. Discussion

The microstructure of the Mg-3Zn-2Ca GC alloy was formed by α grains (solid solution of alloying elements in Mg) with second phase particles Mg_2_Ca and Ca_2_Mg_6_Zn_3_ at the grain boundaries (Figure 1a,b). This alloy was subsequently processed by SCL, SCS and ECAP methods. Grain size and arrangement of second phases were changed by all the applied methods. Mutual features, grain size and network of secondary phases can be found in the microstructure after SLC and SCS processed alloys. Grain size refinement after the SLC and SCS processes was documented; see Table 1 and Figure 2 and Figure 3. Moreover, grains sized approximately 200 μm were found in the microstructure after the SCS process. Observed structural bimodality of SCS alloy was a result of the semisolid processing where only partial liquidizing of the alloy was reached. Obtained small grains were created by the solidification of localized melted metal areas while the large grains were created by the grain coarsening in non-liquidized areas due to high processing temperature. Elongated grains in ECAP direction was found in the ECAPed alloy. Grain size increased (compared with GC alloy) in ECAP direction which was caused by the elevated processing temperature in combination with the severe plastic deformation during the process. 

The HT applied on all Mg-3Zn-2Ca alloys partially dissolved particles of both types. The Mg_2_Ca phase was reshaped into spheres surrounded by a thin shell of the Ca_2_Mg_6_Zn_3_ intermetallic phase. Zn atoms from the Ca_2_Mg_6_Zn_3_ phase diffused into the α solid solution while the Ca atoms were absorbed into the Mg-Ca phase. Smaller and rounded particles in the microstructure were distributed more homogeneously which was reflected in the increase of the ultimate tensile/compression strength and tensile strain to fracture. The HT applied on SCL and SCS alloy completely destroyed the network of particles at grain boundaries. Moreover, Ca_2_Mg_6_Zn_3_ intermetallic phase was completely dissolved during the SCS process. The HT applied before ECAP resulted in more homogeneous distribution of the individual intermetallic phases in the microstructure compared to the direct ECAP of the GC alloy.

Plastic deformation of hexagonal magnesium polycrystals occurs by glide of dislocations and/or twinning. The glide of dislocations with the Burgers vector of $1/3\left[11\bar{2}0\right]$ in the (0001) basal plane is the easiest slip mechanism; it is often called basal slip of <*a*> type dislocations. The critical resolved shear stress (CRSS) is required to activate the glide of <*a*> type dislocations. The yield strength of polycrystals is connected with the CRSS of single crystals with help from the relationship σ_y_ = Mτ_0_, where M is the Taylor orientation factor. The typical feature of the mostly polycrystalline magnesium alloys is limited plasticity at lower temperatures. It is due to the hexagonal close-packed structure of the studied alloy. According to the von Mises criterion for compatible deformation of polycrystals, activity of five independent slip systems is necessary [30] because only two independent slip systems in the basal plane activity of non-basal slip systems are necessary. The slip may run in the prismatic planes by <*a*> dislocation or in prismatic and pyramidal planes by <*a* + *c*> dislocations with the Burgers vector of $1/3\left[11\bar{23}\right]$. The critical resolved yield stress in these non-basal slip systems is at room temperature much higher in comparison with the easy glide of the <*a*> dislocations in the basal plane. Limited mobility of the non-basal dislocations is the reason for the low plasticity of these materials at temperatures up to 200 °C [31]. It is noteworthy that mechanical twinning as the alternative deformation mechanism may be active especially at lower temperatures. The stress necessary for continuous deformation increases with strain up to the maximum stress before it decreases. In the compression tests, a small hump at the beginning of deformation was observed. The stress dependence of the work hardening rate dσ/dε of metallic materials is usually modelled considering two basic types of processes: hardening and softening. It is generally accepted that during deformation the dislocation density changes due to accumulation (storage) of dislocations, interaction between dislocations, static recovery and dynamic recovery. The dislocation accumulation rate depends inversely on the mean free path of dislocations. On the other hand, the mean free path may increase at certain temperatures due to cross slip and/or climb of dislocations [32].

The lowest strength and ductility of the GC alloy measured in tension is caused by the presence of massive precipitates at the grain boundaries and possible casting defects. Moreover, present intermetallic particle distribution is not homogeneous. Partial dissolution of precipitates during the HT decreased the tensile yield stress, σ_02_; on one hand, the ultimate tensile strength, σ_UTS_, increased. Observed dissolution of the secondary phases situated at the grain boundaries and their weakening partially decreased stress screening effect at the grain boundaries and led to the improved ductility. On the other hand, increased concentration of solute atoms increased solid solution hardening in the matrix. The increase of the stress necessary for dislocation motion on the slip plane is proportional to the solute concentration as Δσ ∝ c^2/3^ [33].

Tensile/compression test results show positive influence of grain refinement on mechanical properties of the alloy prepared by SCL and SCS methods. σ_UT(C)S_ and elongation to the fracture were improved. The following HT resulted in further improvement of the σ_UTS_ and elongation of the fracture strain. Complete dissolution of hard Ca_2_Mg_6_Zn_3_ phase after SCS + HT was a reason for lower strength and higher elongation to fracture compared to the SCL alloy. Higher elongation to fracture of materials after the HT compared to the cast alloys (Table 3) may be explained by an increase of the dislocation slip length due to partial dissolution of particles.

The ECAPed alloy microstructure consists of elongated grains oriented into processing direction surrounded by the intermetallic phases present at the grain boundaries. The ECAP treatment resulted in the significant increase of the yield stress and ultimate tensile strength. The obtained tensile strength characteristics were almost doubled compared with the values for the GC alloy. The value of the tensile strain to fracture of the GC alloy was increased by the ECAP; however, the influence was not so significant as in the case of SCL and SCS alloys combined with the heat treatment. Compression strength, yield stress and ultimate tensile strength were increased by the ECAP compared to the GC alloy, while the strain to fracture decreased. The HT applied before ECAP resulted in the more homogeneous distribution of the individual intermetallic phases compared to the direct the ECAP of the CG alloy. The missing net of intermetallic phases resulted in the increased tension characteristics; however, in the case of compression characteristics, only the yield stress was increased while the ultimate compression strength and the strain to fracture were even lower than the values measured for the CG alloy. Differences in tension and compression behavior can be explained by texture of the ECAPed alloy. Crystallographic textures can also change the value of the yield stress, as for instance in the case of extruded magnesium materials deformed in the extrusion direction [34]. A similar texture can be considered for the ECAPed alloy. A great difference between the yield stress obtained in tension and compression was observed for the ECAPed alloy—see Table 3 and Table 4. The shapes of curves measured in tension and compression differs substantially (Figure 9). While the true stress–strain curve obtained in tension is flat, the curve observed in compression has a local maximum at a strain of 15%–20%. It is known that extruded magnesium alloys exhibit pronounced texture where basal (0001) planes are oriented parallel to the extrusion direction [35]. Specimens with such texture are not able to deform by twinning in the tensile experiment. On the other hand, tensile twins $\left\{10\bar{1}2\right\}\;\langle 10\bar{1}1\rangle$ may be activated in the compression experiment. Such twinning causes a misorientation of 86.3° between the twinned and the untwinned lattice [35]. The tensile deformation is realized at the beginning by the motion of <*a*> ($\left\langle 11\bar{2}0\right\rangle$) dislocations in the basal and prismatic planes, while deformation in the c-axis is provided by the activity of <*c* + *a*> ($\left\langle 11\bar{2}\bar{3}\right\rangle$) dislocations. The high Schmid factor for twinning is the reason why twinning in compression is activated at relatively low stresses. The twinning deformation is exhausted at the plastic strain of several percent and further deformation continues by the dislocation motion in the reoriented grains. Contribution of twinning to the whole deformation is relatively low, but not negligible. On the other hand, twin boundaries representing the non-dislocation obstacles for dislocation motion are reason for the high strain hardening in the compression test. It is interesting to note that the yield stresses obtained in the ECAPed alloy are relatively high, whereas the grain size achieved in this material has the biggest value. The explanation can be found in high dislocation density in specimens after the severe plastic deformation in the ECAP tool and in the fine distribution of secondary phases in the matrix.

In the case of SCS and ECAP alloys, serrated flow was observed in the true stress–strain curves measured in compression. Such behavior was observed in several magnesium alloys. Trojanová et al. [36] observed the Portevin-Le Châtelier (PLC) effect in the AZ91 (Mg-9 wt%Al-1 wt%Zn) alloy after homogenization treatment in tension and compression. It was estimated that the achievement of some critical strain step is necessary for the gaining of the PLC effect. This critical strain was very probably not achieved in tension due to small plasticity of SCS and ECAPed alloys and it is the reason why the PLC effect in tension was not found for the Mg-3Zn-2Ca alloy. The occurrence of the PLC effect needs some free solute atoms which are able to move with the diffusional mechanism [37]. In spite of many attempts, there is no generally accepted model for the unstable plastic deformation. A sudden collective breakaway of dislocations piled up in front of dislocation tangles and their movement on large distances may be assumed as a main reason for observed stress drops on the stress–strain curves. According to [37], glide in the primary slip system results in the formation of stable dislocation pile-ups and associated stress concentrations. The difficulty in activating prismatic slip makes the pile ups formed on the basal planes very stable. The stress concentrations are relaxed by a long-range correlated dislocation motion (cross slip) of a dislocation group in the secondary (prismatic) slip system. Such dislocation motion is allowed only for screw dislocations. The edge components of dislocation loops remain in the original slip plane as forest dislocations for dislocation motion in the secondary slip planes. Cores of dislocations waiting at obstacles during thermally activated motion in the slip plane may be occupied by dissolute atoms, which are movable due to pipe diffusion. During the PLC flow, a drop in the load is possible if the strain rate during the formation of the slip bands exceeds the strain rate imposed by the testing machine. 

## 6. Conclusions

The influence of different processing techniques followed by heat treatment on mechanical properties of the Mg-3Zn-2Ca alloy was studied. Microstructure observations and deformation tests showed these main results:

1. The main microstructure feature of the gravity cast alloy was dendritic in structure with a network of intermetallic phases, namely Ca_2_Mg_6_Zn_3_ and Mg_2_Ca. The squeeze cast alloy in the liquid state resulted in the fine microstructure composed of fine α grains. Squeeze cast alloy in the semisolid state exhibited the bimodal microstructure resulting from semisolid processing where only partial liquidizing of the alloy was reached. Original microstructure with more equiaxial grains was changed during the equal channel angular pressing (ECAP) procedure into the microstructure with elongated grains in the extrusion direction. Applied heat treatment refined the microstructure and partially dissolved particles in all alloys. The heat treatment transmitted the spheroidization and partial dissolution of intermetallic phases accompanied by the change of their chemical composition. In the case of squeeze cast alloy in the semisolid state, the complete dissolution of Ca_2_Mg_6_Zn_3_ intermetallic particles was observed.
2. Low plasticity of all the samples observed in tension was improved by the heat treatment due to the microstructure homogenization and partial dissolution of precipitates.
3. Basal texture developed during the equal channel hot extrusion activates the twinning at a low degree of deformation which results in a comparably lower yield stress in the compressive test.
4. Serrated flow, observed in the compression tests of squeeze cast semisolid alloy (SCS) and ECAPed samples, is a consequence of interactions between solute atoms and movable dislocations (i.e., the Portevin-Le Châtelier effect).

## Figures and Tables

**Table 1:** Average grain size of the alloy processed by various methods.

Processing Method	GC	SCL	SCS	ECAP
average grain size (μm)	40.7 ± 5.9	9.6 ± 0.7	bimodal 9.6 ± 0.7 up to 200	84.3 ± 0.3 ECAP direction 30.5 ± 2.6 perpendicular d

**Table 2:** Chemical composition of the alloy processed by various methods.

Alloy State	Phase	Element (wt %)			Element (at %)		
		Mg	Ca	Zn	Mg	Ca	Zn
**GC**	hcp Mg	98.3	0.4	1.3	99.2	0.3	0.5
	Mg_2_Ca	70.0	24.8	5.1	80.3	17.4	2.2
	Ca_2_Mg_6_Zn_3_	62.9	10.5	26.6	79.4	8.1	12.5
**GC + HT**	hcp Mg	97.3	0.5	2.2	98.9	0.3	0.8
	Mg_2_Ca	56.8	37.9	5.3	69.5	28.1	2.4
	Ca_2_Mg_6_Zn_3_	71.3	7.2	21.6	85.2	5.2	9.6
**SCL**	hcp Mg	98.7	0.4	1.0	99.4	0.2	0.4
	Mg_2_Ca	80.0	15.2	4.8	87.9	10.1	1.9
	Ca_2_Mg_6_Zn_3_	66.2	11.2	22.6	81.3	8.3	10.5
**SCL + HT**	hcp Mg	97.5	0.5	2.0	99.0	0.3	0.7
	Mg_2_Ca	59.1	36.3	4.6	71.4	26.6	2.1
	Ca_2_Mg_6_Zn_3_	72.0	8.6	19.4	85.3	6.2	8.5
**SCS**	hcp Mg	99.1	0.2	0.7	99.6	00.1	0.3
	Mg_2_Ca	75.0	19.1	6.0	84.5	13.0	2.5
	Ca_2_Mg_6_Zn_3_	62.7	11.4	26.0	79.0	8.7	12.2
**SCS + HT**	hcp Mg	97.7	0.4	1.9	99.0	0.3	0.7
	Mg_2_Ca	57.9	36.9	5.2	70.4	27.3	2.4
	Ca_2_Mg_6_Zn_3_	not present in the microstructure			not present in the microstructure		

**Table 3:** Mechanical characteristics of the Mg-3Zn-2Ca alloy processed by different methods obtained in tension: yield stress, σ_02_, ultimate tensile strength, σ_UTS_, and strain to fracture, e.

Alloy State	σ_02_ (MPa)	σ_UTS_ (MPa)	e (%)
GC	89.8	101.0	0.4
GC + HT	87.8	126.0	2.0
SCL	79.7	134.8	0.9
SCL + HT	74.2	144.1	3.3
SCS	67.0	110.6	1.2
SCS + HT	67.6	133.5	4.4
ECAP	166.1	206.4	1.1
HT + ECAP	173.5	223.0	2.4

**Table 4:** Mechanical characteristics of Mg-3Zn-2Ca alloy processed by different methods obtained in compression: yield stress, σ_02_, ultimate compression strength, σ_UCS_, and strain to fracture, ε_B_.

Alloy State	σ_02_ (MPa)	σ_UCS_ (MPa)	ε_B_ (%)
GC	83.4	234.6	15.5
GC + HT	80.1	334.9	13.7
SCL	84.5	243.2	15.2
SCL + HT	85.3	273.2	15.0
SCS	71.2	219.5	18.4
SCS + HT	73.4	220.2	18.3
ECAP	119.6	296.8	10.8
HT + ECAP	131.6	299.6	9.7
